# Trends and outcomes of cardiac arrest and extracorporeal membrane oxygenation during the COVID-19 pandemic in the United States

**DOI:** 10.1371/journal.pone.0334896

**Published:** 2025-10-16

**Authors:** Joseph Hadaya, Shayan Ebrahimian, Nam Yong Cho, Shineui Kim, Peyman Benharash

**Affiliations:** Division of Cardiac Surgery, Department of Surgery, David Geffen School of Medicine, University of California, Los Angeles, California, United States of America; Azienda Ospedaliero Universitaria Careggi, ITALY

## Abstract

**Background:**

The COVID-19 Pandemic challenged the healthcare system worldwide, but its effect on outcomes of cardiac arrest (CA) and extracorporeal membrane oxygenation (ECMO) use are understudied. We examined trends in CA, ECMO use and survival, and evaluated the impact of receiving care during the COVID-19 Pandemic on outcomes following CA. We also evaluated the impact of COVID-19 infection on outcomes following CA.

**Methods:**

Adults with out-of-hospital (OHCA) or in-hospital cardiac arrest (IHCA) were identified in the 2016−2020 National Inpatient Sample. For primary analysis, CA patients without COVID-19 were divided into Pre-Pandemic and Pandemic time-periods. For secondary analysis, CA patients treated during the Pandemic time-period were divided by COVID-19 infection status. Generalized linear models were used to evaluate associations between Pandemic time-period or COVID-19 infection with in-hospital mortality.

**Results:**

Of 1,320,020 non-COVID-19 CA patients, 19.1% were managed during the Pandemic. From 2016−2019, CA incidence increased from 696 to 771 per 100,000 hospitalizations, and disproportionately increased to 1,023 per 100,000 hospitalizations by the end of 2020. Mortality for IHCA was stable prior to the Pandemic, but increased from 67.4% to 75.4% by the end of 2020, while mortality for OHCA was stable. ECMO use increased from 2016 to 2019 for OHCA and IHCA, declined during the second quarter of 2020, and recovered to pre-Pandemic levels by the end of 2020. After risk-adjustment, care during the Pandemic was associated with 1.2-fold greater odds of mortality after CA for non-COVID-19 patients. Among 277,975 patients experiencing CA during the Pandemic, 19.6% had concomitant COVID-19 infection. After risk-adjustment, COVID-19 infection was associated with 3.9-fold greater odds of mortality after CA.

**Conclusion:**

CA incidence and mortality increased during the COVID-19 Pandemic, while ECMO use declined, emphasizing the need to improve care of CA and ECMO patients. COVID-19 patients with CA had dismal outcomes, suggesting no role for ECMO in this population.

## Introduction

The incidence of cardiac arrest has increased over the last decade in the United States, and accounts for approximately 400,000 deaths annually [1]. Modest improvements in cardiac arrest survival over the last two decades have been, in part, attributable to standardized cardiopulmonary resuscitation protocols, provider education and awareness, as well as more efficient pre-hospital care systems [2]. Nonetheless, survival with intact neurological function remains dismal at 7–12% among all patients suffering from cardiac arrest [1,3]. Extracorporeal membrane oxygenation (ECMO) has been increasingly utilized as rescue therapy for refractory cardiac arrest [4–6]. Institution of ECMO during cardiac arrest or following return of spontaneous circulation allows for rapid normalization of end-organ perfusion and may facilitate interventions and medical therapies to address the underlying cause of the arrest.

Prior studies have demonstrated a significant rise in the utilization of ECMO among patients in cardiac arrest [4,6]. Three recent randomized controlled trials of ECMO for out-of-hospital cardiac arrest (OHCA) have yielded mixed results. One study was terminated early due to the superiority of ECMO, a second was stopped for lack of efficacy, and a third demonstrated no difference between extracorporeal and conventional cardiopulmonary resuscitation [7–9]. With the dawn of the Coronavirus Disease 2019 (COVID-19) pandemic, many institutions transiently limited their ECMO programs or altered selection criteria, while pre-hospital/ hospital systems modified their practices to ensure the safety of healthcare workers [10,11]. Data on the impact of the COVID-19 pandemic on the incidence of cardiac arrest, the use of ECMO, and associated outcomes across the United States, remains limited.

In the present study, we evaluated recent trends in cardiac arrest, incident ECMO utilization, and survival to discharge using a nationally representative cohort. We subsequently compared characteristics and outcomes of patients without COVID-19 who experienced cardiac arrest before and after the COVID-19 pandemic, and identified factors associated with use of ECMO. Finally, we examined outcomes of cardiac arrest patients with a concomitant diagnosis of COVID-19 relative to all others. We hypothesized an increase in the incidence of cardiac arrest during the COVID-19 pandemic with an associated decrease in ECMO use and inferior survival for both COVID-19 and non-COVID-19 patients during the pandemic.

## Methods

### Data source and study approval

This was a retrospective cohort study using the National Inpatient Sample (NIS). The NIS is maintained as part of the Healthcare Cost and Utilization Project, a federal-state-industry initiative to evaluate inpatient healthcare utilization, access, costs, and outcomes across the United States. The NIS is the largest, all-payer database and provides accurate estimates for over 97% of US inpatient hospitalizations. [12] The study was deemed exempt from full review by our university’s Institutional Review Board due to the deidentified nature of the NIS. Individual patients can not be identified nor contacted for consent due to the structure of NIS. Deidentified data was accessed from February 1, 2024 to August 18, 2024 for the purpose of the present analysis.

### Study population, variables, and outcomes

All adults who experienced out-of-hospital (OHCA) or in-hospital cardiac arrest (IHCA) from 2016 to 2020 were identified using relevant International Classification of Diseases, Tenth Edition (ICD-10) codes (S1 Table). Patients with both sets of codes were classified under IHCA as they had continued to receive chest compressions during hospitalization. Patients with missing data for demographics or key outcomes were excluded from further study.

**Fig 1:**
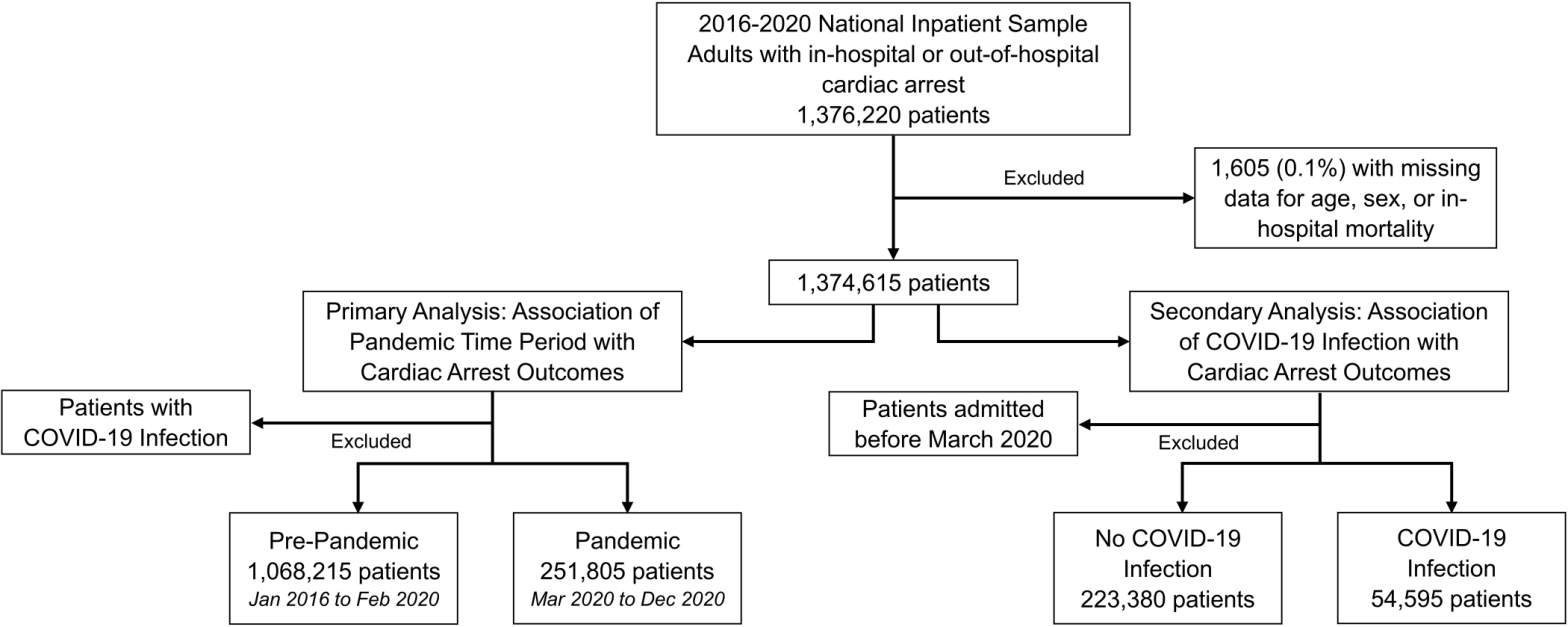
Flow diagram of patients included in primary and secondary analyses.

Patients were subsequently divided into two cohorts to address the study questions (Fig 1). To evaluate the impact of the COVID-19 pandemic on outcomes of cardiac arrest, we divided patients without a diagnosis of COVID-19 infection into a pre-pandemic time period, defined as January 2016 to February 2020, and a pandemic time period, defined as March 2020 to December 2020. Patients with a diagnosis of COVID-19 were specifically excluded from this cohort, as we hypothesized the outcomes of this group would differ from all others. To evaluate the impact of acute COVID-19 infection on outcomes of cardiac arrest, our second cohort was comprised solely of patients treated during the pandemic time period (March 2020 to December 2020). This cohort was divided into two groups based on the presence or absence of acute COVID-19 infection, ascertained using ICD-10 diagnosis codes (S1 Table).

Patient and hospital characteristics are reported as defined by the NIS and included age, sex, primary insurer, zip code-adjusted income quartile, hospital region, and hospital size [12]. The Elixhauser Comorbidity Index, a weighted score encompassing over 30 chronic medical conditions, was used to evaluate the overall burden of chronic disease [13]. Patients with ventricular tachycardia (VT) or ventricular fibrillation (VF) were identified using ICD-10 codes, while those lacking these codes were considered to have asystole or pulseless electrical activity (PEA), as these diagnoses do not carry specific codes. Costs were calculated from charges using cost-to-charge ratio files provided by HCUP, and adjusted for inflation to 2020. Outcome measures included mortality, length of stay, and hospitalization costs.

The primary aim of the study was to examine recent trends in cardiac arrest and associated ECMO use and survival, and evaluate the impact of receiving care during the COVID-19 pandemic on outcomes following cardiac arrest across the United States. We secondarily aimed to evaluate the impact of acute COVID-19 infection on outcomes following cardiac arrest.

### Statistical analysis

Categorical variables are reported as percentages and compared using the chi-squared test. Continuous variables are reported as means with standard deviation or medians with interquartile range, and compared using the adjusted Wald test or Wilcoxon rank sum test, as appropriate. A rank-based nonparametric test was used to study temporal changes (Cuzick’s) [14]. Generalized linear models were used to evaluate the association between the pandemic time period and outcomes measures, or acute COVID-19 infection and outcome measures. Model covariates were selected using the least absolute shrinkage and selection operator (LASSO), a regression algorithm commonly employed in large data sets to minimize model overfitting and improve out-of-sample model validity [15]. Models were subsequently evaluated using the area under the receiver operating characteristics curve and Bayesian information criteria. Regression outputs were reported as adjusted odds ratio or beta-coefficient with 95% confidence interval (CI). Statistical significance was set at a two-sided α < 0.05. All statistical analysis was performed using Stata 16.0 (StataCorp, College Station, Texas).

## Results

### Trends in cardiac arrest, ECMO use, and mortality among non-COVID-19 patients

**Fig 2:**
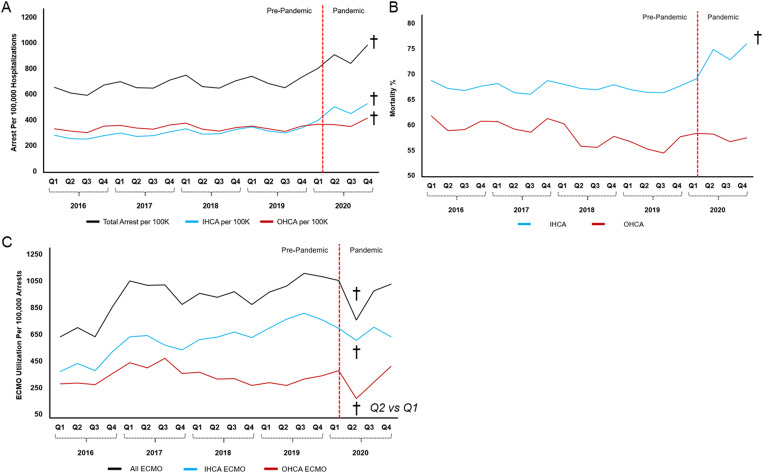
Trends in the incidence of Cardiac Arrest (A), Mortality following Cardiac Arrest (B), and ECMO Utilization for Cardiac Arrest (C). Incidence in cardiac arrest normalized to hospitalizations, as the National Inpatient Sample estimates 97% of United States hospitalizations. Red bar divides Pre-Pandemic and Pandemic time-periods. †p < 0.05.

From 2016 to 2019, the incidence of cardiac arrest increased from 696 to 771 per 100,000 hospitalizations, and further disproportionately increased to 1,023 per 100,000 hospitalizations by quarter 4 of 2020 (p < 0.001). This was evident for both in-hospital and out-of-hospital cardiac arrest (Fig 2A). Prior to the COVID-19 pandemic, mortality for IHCA was relatively stable (68.4% in 2016 to 67.4% in 2019) but increased to 75.4% by quarter 4 of 2020 for Non-COVID-19 patients (p < 0.001). Mortality for OHCA modestly decreased from 61.7% to 58.4% from 2016 to 2019, and remained stable at 57.5% in 2020 (Fig 2B). ECMO use among non-COVID-19 patients significantly increased from 2016 to 2019 for both OHCA and IHCA (p < 0.001), declined during the second quarter of 2020, and recovered to pre-pandemic levels by the end of 2020 (Fig 2C).

### Characteristics and outcomes of patients experiencing cardiac arrest before and after the COVID-19 pandemic

Of an estimated 1,320,020 patients who experienced cardiac arrest, 19.1% (251,805) were managed during or after the COVID-19 pandemic. Patient and hospital characteristics, stratified by pre-pandemic or post-pandemic time frame, are reported in Table 1. Patients managed during the COVID-19 pandemic were less commonly female (41.4% vs 42.8%, p < 0.001), of comparable age (64.2 vs 64.4, p = 0.17), and were more commonly of non-White race. Following the COVID-19 pandemic, cardiac arrests were more commonly in-hospital (55.8% vs 47.4%, p < 0.001) and more commonly asystole or PEA (78.2% vs 76.7%, p < 0.001). Coronary angiography was used less frequently employed following the pandemic (12.2% vs 14.7%, p < 0.001), while ECMO use remained steady (0.9% vs 0.9%). The overall burden of chronic conditions, as defined by the Elixhauser Comorbidity Index, was greater following the COVID-19 pandemic (5.5 vs 5.4, p < 0.001), though most specific chronic conditions were comparable between groups.

**Table 1:** Patient and hospital characteristics for non-COVID-19 cardiac arrest patients treated from 2016-2020 stratified by Pre-Pandemic and Pandemic time periods. ECMO, extracorporeal membrane oxygenation; PEA, pulseless electrical activity; SD, standard deviation; VF, ventricular fibrillation; VT, ventricular tachycardia.

	Pre-Pandemic n = 1,068,215	Pandemic n = 251,805	P-value
Age, years (mean ± SD)	64.4 ± 18.4	64.2 ± 17.7	0.17
Female (%)	42.8	41.5	<0.001
Income Quartile (%)			0.03
76-100^th^ Percentile	17.5	16.5	
51-75^th^ Percentile	22.4	21.4	
26-50^th^ Percentile	25.9	26.7	
0-25^th^ Percentile	34.1	35.3	
Race (%)			<0.001
White	63.6	59.0	
Black	19.6	51.3	
Hispanic	9.8	12.1	
Asian and Pacific Islander	3.1	3.1	
Other race	3.9	4.5	
Primary Insurer (%)			<0.001
Private	18.3	18.5	
Medicare	59.9	57.6	
Medicaid	14.3	15.6	
Other payer	3.1	3.6	
Uninsured	4.4	4.7	
Comorbidities (%)			
Cerebrovascular disease	11.8	11.1	<0.001
Chronic pulmonary disease	27.3	25.4	<0.001
Cirrhosis	3.8	4.2	<0.001
Congestive heart failure	43.3	42.9	0.12
Coronary artery disease	37.3	34.4	<0.001
End-stage renal disease	14.6	14.7	0.80
Hypertension	67.4	68.0	0.12
Liver disease	17.3	19.0	<0.001
Malignancy	10.0	9.6	0.053
Myocardial infarction	26.8	26.5	0.35
Pulmonary hypertension	12.6	12.5	0.64
Pulmonary embolism	4.5	5.0	<0.001
Elixhauser Comorbidity Index (mean ± SD)	5.4 ± 2.4	5.5 ± 2.3	<0.001
Arrest Characteristics			
Location (%)			<0.001
Out-of-hospital cardiac arrest	52.6	44.2	
In-hospital cardiac arrest	47.4	55.8	
Rhythm (%)			<0.001
VT or VF	23.2	21.8	
Asystole or PEA	76.8	78.2	
Procedures (%)			
ECMO	0.9	0.9	0.86
Coronary angiography	14.7	12.2	<0.001
Hospital Characteristics			
Bed size (%)			0.002
Small	17.0	20.3	
Medium	29.8	29.9	
Large	53.2	49.8	
Region (%)			0.76
Northeast	15.6	15.6	
Midwest	20.8	19.7	
South	43.2	44.3	
West	20.4	20.4	
Teaching hospital (%)	72.8	76.2	0.002

Unadjusted mortality rates following cardiac arrest were significantly greater during the pandemic compared to the prior era (66.8% vs 62.5%, p < 0.001, Table 2). In addition, hospitalization costs were greater following the COVID-19 pandemic than before ($23,700 vs $21,800, p < 0.001, Table 2), as was hospital length of stay (median 5 vs 4 days, p < 0.001). After adjustment for patient, hospital, and arrest characteristics, care received following the COVID-19 pandemic was associated with greater odds of mortality following cardiac arrest (adjusted odds ratio, AOR, 1.16, 95% CI 1.12–1.19, S2 Table), as well as an incremental increase in length of stay by 0.3 days (95% CI 0.1–0.5) and costs by $3,000 (95% CI 1,800−4,300, Table 2).

**Table 2:** Unadjusted and risk-adjusted outcomes among non-COVID-19 cardiac arrest patients treated from 2016−2020, stratified by Pre-Pandemic and Pandemic time-periods. CI, confidence interval; COVID-19, Coronavirus 2019; ECMO, extracorporeal membrane oxygenation; IQR, interquartile range.

	Pre-Pandemic	Pandemic	P-value	Adjusted Odds Ratio or ß (95% CI)	P-value
	(n = 1,068,215)	(n = 251,805)			
In-Hospital Mortality (%)	62.5	66.8	<0.001	1.16 (1.12-1.19)	<0.001
ECMO Utilization (%)	0.9	0.9	0.86	0.99 (0.85-1.16)	0.54
Length of Stay, days (median, IQR)	4 (1-11)	5 (2-12)	<0.001	0.27 (0.08-0.45)	0.005
Hospitalization Costs, $1,000s (median, IQR)	21.8 (10.2-44.8)	23.7 (11.2-48.9)	<0.001	3.0 (1.8-4.3)	<0.001

**Fig 3:**
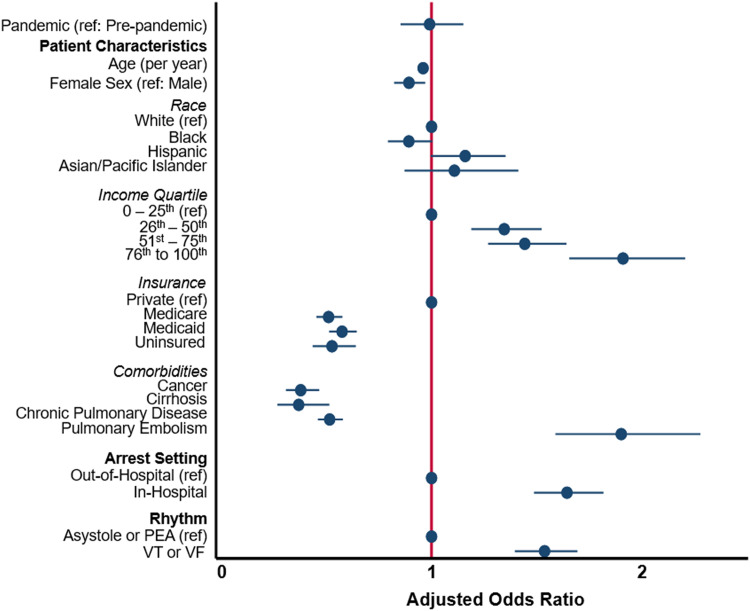
Factors associated with ECMO utilization among non-COVID-19 cardiac arrest patients treated from 2016-2020. Estimates represent adjusted odds ratio and 95% confidence interval. Additional factors including congestive heart failure, myocardial infarction, history of cerebrovascular disease, history of renal disease, and others, omitted from display for but contribute to risk-adjusted model.

Following risk-adjustment, there was no association between the pandemic time period and ECMO use (Fig 3). Factors associated with ECMO use included younger age, private insurance, increasing income, and care in the Northeastern region of the United States. Arrest characteristics associated with ECMO use included IHCA, rhythm of VF/VF relative to PEA or asystole. Certain acute medical conditions such as myocardial infarction, pulmonary embolism, and heart failure were associated with greater ECMO use, while other chronic conditions including cirrhosis, malignancy, cerebrovascular disease, and end stage renal disease were associated with reduced use of ECMO (S3 Table).

### Characteristics and outcomes of COVID-19 versus Non-COVID-19 patients experiencing cardiac arrest

To evaluate the impact of acute COVID-19 infection on outcomes following cardiac arrest, we compared characteristics and outcomes of COVID-19 patients to non-COVID-19 patients who developed cardiac arrest during the pandemic (March 2020 and later). Of 277,975 patients experiencing cardiac arrest during the pandemic, 54,595 (19.6%) had a concomitant diagnosis of COVID-19 infection (Table 3). COVID-19 patients were less commonly female (38.4% vs 42.0%, p < 0.001), older (65.7 vs 61.6 years, p < 0.001), more commonly Black (24.6% vs 20.5%) or Hispanic (26.9% vs 10.2%, p < 0.001), and more commonly in the lowest income quartile (41.5% vs 34.6%, p < 0.001). In-hospital cardiac arrest was modestly more common among COVID-19 patients compared to others (52.1% vs 50.2%, p < 0.001), as were PEA or asystole arrests (88.0% vs 77.1%, p < 0.001). Coronary angiography was less commonly used among COVID-19 patients (1.6% vs 13.6%, p < 0.001) while ECMO use was comparable (0.9% vs 1.0%). COVID-19 patients were more commonly managed in the Northeast and at small or medium-sized hospitals. The overall burden of comorbidities was lower for COVID-19 patients relative to non-COVID-19 (Elixhauser Comorbidity Index 5.17 vs 5.21, p < 0.001), as were specific conditions including myocardial infarction, chronic pulmonary disease, liver disease, and malignancy (Table 3).

**Table 3:** Patient and hospital characteristics solely among those experiencing cardiac arrest during the Pandemic time period, stratified by presence of acute COVID-19 diagnosis. ECMO, extracorporeal membrane oxygenation; PEA, pulseless electrical activity; SD, standard deviation; VF, ventricular fibrillation; VT, ventricular tachycardia.

	Non-COVID-19 n = 223,380	COVID-19 n = 54,595	P-value
Age, years (mean ± SD)	61.6 ± 19.3	65.7 ± 15.0	<0.001
Female (%)	42.0	38.4	<0.001
Income Quartile (%)			<0.001
76-100^th^ Percentile	17.0	12.7	
51-75^th^ Percentile	21.6	20.1	
26-50^th^ Percentile	26.8	25.7	
0-25^th^ Percentile	34.6	41.5	
Race (%)			<0.001
White	62.0	38.4	
Black	20.5	24.6	
Hispanic	10.2	26.9	
Asian and Pacific Islander	3.0	3.6	
Other race	4.3	6.5	
Primary Insurer (%)			<0.001
Private	18.4	20.3	
Medicare	57.6	57.9	
Medicaid	15.7	13.7	
Other payer	3.5	4.3	
Uninsured	4.8	3.8	
Comorbidities (%)			
Cerebrovascular disease	11.4	9.4	<0.001
Chronic pulmonary disease	25.7	22.4	<0.001
Cirrhosis	4.5	1.9	<0.001
Congestive heart failure	44.3	29.8	<0.001
Coronary artery disease	35.2	27.1	<0.001
End-stage renal disease	14.6	14.1	0.09
Hypertension	67.1	74.3	<0.001
Liver disease	19.7	13.6	<0.001
Malignancy	10.4	3.5	0.053
Myocardial infarction	27.4	18.4	<0.001
Pulmonary hypertension	12.8	9.6	<0.001
Pulmonary embolism	4.9	5.3	0.10
Elixhauser Comorbidity Index (mean ± SD)	5.2 ± 2.4	5.2 ± 2.1	<0.001
Arrest Characteristics			
Location (%)			<0.001
Out-of-hospital cardiac arrest	49.8	47.9	
In-hospital cardiac arrest	50.2	52.1	
Rhythm (%)			<0.001
VT or VF	22.9	12.0	
Asystole or PEA	77.1	88.0	
Procedures (%)			
ECMO	0.9	1.0	0.63
Coronary angiography	13.6	1.6	<0.001
Hospital Characteristics			
Bed size (%)			<0.001
Small	19.8	23.8	
Medium	29.7	32.9	
Large	50.5	43.3	
Region (%)			<0.001
Northeast	15.1	20.4	
Midwest	20.1	15.0	
South	44.6	44.5	
West	20.2	20.1	
Teaching hospital (%)	76.4	73.5	<0.001
Admission Month in 2020 (%)			<0.001
March	10.3	5.5	
April – June	27.4	28.5	
July – September	29.4	25.7	
October – December	32.8	40.3	

Unadjusted mortality rates were significantly greater for COVID-19 patients experiencing cardiac arrest compared to non-COVID-19 patients (88.6% vs 63.4%, p < 0.001, Table 4). Compared to non-COVID-19 patients, COVID-19 patients had greater length of stay (9 vs 4 days, p < 0.001) and greater associated hospitalization costs ($29,800 vs $23,000, p < 0.001). In 2020, COVID-19 infection was associated with greater odds of ECMO use (1.8, 95% CI 1.4–2.4). Following risk-adjustment, COVID-19 infection was associated with 3.9 fold greater odds of mortality (95% CI 3.7–4.2) and a risk-adjusted increase in length of stay by 4.0 days (95% CI 3.7–4.4) and costs by $9,700 (95% CI 8,100−11,400).

**Table 4:** Unadjusted and risk-adjusted outcomes among patients treated during the Pandemic time period stratified by presence of COVID-19 diagnosis. CI, confidence interval; COVID-19, Coronavirus 2019; ECMO, extracorporeal membrane oxygenation; IQR, interquartile range.

	Non-COVID-19	COVID-19	P-Value	Adjusted Odds Ratio or ß (95% CI)	P-Value
	(n = 223,380)	(n = 54,595)			
In-hospital Mortality (%)	63.7	88.6	<0.001	3.94 (3.67-4.23)	<0.001
ECMO Utilization (%)	0.9	0.9	0.63	1.83 (1.39-2.43)	<0.001
Length of Stay, days (median, IQR)	4 (1-11)	9 (4-17)	<0.001	4.04 (3.71-4.37)	<0.001
Hospitalization Costs, $1,000s (median, IQR)	23.0 (10.8-47.5)	29.8 (13.9-60.2)	<0.001	9.7 (8.1-11.4)	<0.001

## Discussion

Despite advances in the resuscitation and management of cardiac arrest patients, survival to hospital discharge remains poor. In the present work, we noted the incidence of cardiac arrest among non-COVID-19 patients to have significantly increased during the COVID-19 pandemic. In contrast to the trends noted over the last decade, mortality after cardiac arrest rose significantly during the pandemic. The use of ECMO and coronary angiography initially declined during the COVID-19 pandemic but recovered to pre-pandemic levels, while mortality remained elevated. Cardiac arrest characteristics significantly differed among COVID-19 and non-COVID-19 patients, while the former experienced significantly inferior outcomes. Several of these findings warrant further discussion.

Prior reports on cardiac arrest during the COVID-19 pandemic have reported mixed findings. In Germany, Roedl et al. reported a trivial increase in the incidence of in-hospital cardiac arrest among all-comers in a three-month period during the COVID-19 pandemic, with rates increasing from 4.6 to 6.6 per 1,000 hospitalizations [16]. On the contrary, Tong et al. reported a modest decrease in in-hospital cardiac arrest rates among inpatients in Hong Kong who did not carry a diagnosis of acute COVID-19 infection during the first year of the pandemic [17]. For out-of-hospital cardiac arrest, several studies suggest a transient increase in rates in the United States and worldwide during the pandemic, though limited data differentiates patients with acute COVID-19 from those not infected. A study of the Los Angeles County emergency medical services registry demonstrated an increase in emergency medical services responses to OHCA by 21% for the first two months of the pandemic [18]. Interestingly, provider impression for ST-elevation myocardial infarction decreased by 7% as did the rates of defibrillation use or transport to hospital, suggesting that the nature of these arrests was different than the pre-pandemic period [18]. In our analysis, we found an approximately 17% increase in the incidence of cardiac arrest during the pandemic compared to the preceding year among patients without COVID-19. This was primarily driven by IHCA, while OHCA remained relatively stable (1% increase), though our estimates for OHCA are likely an underestimate as patients must be transported and survive to hospitalization to be included in this database. While the nature of increased cardiac arrests during the pandemic period among non-COVID-19 patients is unknown, several studies have hypothesized reduced outpatient care utilization, delayed presentation of acute or chronic medical illness due to concern for exposure to COVID-19, and reallocation of healthcare resources as putative factors [19,20].

Mortality following cardiac arrest has gradually declined over the last two decades [1,2]. Several studies have reported an increase in mortality for both OHCA and IHCA during the COVID-19 pandemic, though few distinguish patients with acute COVID-19 infection, particularly for OHCA. Lai et al examined 5,325 OHCA cases in New York City during the first two months of the COVID-19 pandemic and found rates of return of spontaneous circulation to have decreased by nearly 50%, with a greater proportion of patients presenting with a non-shockable rhythm [21]. Although this study could not distinguish COVID-19 from non-COVID-19 patients, the rise of OHCA cases mirrored emergency medical service calls for COVID-19 symptoms, suggesting that OHCA mortality is partly attributable to COVID-19 infection [21]. In a single institution analysis of 125 in-hospital cardiac arrests during the early pandemic period, Miles et al. reported a reduction in absolute survival by 10% among all hospitalized patients, including those with acute COVID-19 infection [22]. Concomitant with an increase in cardiac arrest in our study, we found a substantial rise in mortality following cardiac arrest during the pandemic among non-COVID-19 patients. Compared to the year prior to the pandemic, mortality following IHCA increased by approximately 12% while OHCA remained stable. As the primary analysis contained solely patients without COVID-19, our data suggests a major deterioration in cardiac arrest outcomes temporally associated with the COVID-19 pandemic. Future work to examine whether these trends continue, identify drivers of worse outcomes, and implement protocols to continue to improve cardiac arrest care are needed.

Extracorporeal membrane oxygenation has been increasingly utilized in cases of cardiopulmonary resuscitation or refractory shock. In the absence of a universal consensus, current guidelines from the Extracorporeal Life Support Organization recommend considering ECMO in patients likely to survive with favorable neurologic outcomes and in the absence of life-limiting medical conditions [23]. In our analysis of cardiac arrest patients across the United States, we noted ECMO utilization to have declined during the first phase of the pandemic, recovering to pre-pandemic levels by the end of 2020. We found several factors to be associated with ECMO utilization including a rhythm of VT or VF and the presence of acutely reversible conditions such as a myocardial infarction or pulmonary embolism. Similarly, chronic medical conditions such as malignancy, end stage renal disease, and cirrhosis were associated with reduced odds of ECMO deployment. Similar observations were noted when examining the use of coronary angiography among patients with cardiac arrest, which also declined in use during the COVID-19 pandemic. The transient decrease in ECMO or coronary angiography utilization may be related to transient suspension of ECPR programs, institutional efforts to initially reduce exposure of healthcare providers to COVID-19, or to changes in arrest characteristics, though our database is limited in identifying these factors [11,24].

While outcomes of cardiac arrest and ECMO for cardiac arrest have been previously described at institutional and national levels for all-comers, limited large-scale data exists regarding cardiac arrest outcomes of COVID-19 patients. In our analysis, we found patients with acute COVID-19 infection to face nearly four-fold greater odds of in-hospital mortality compared to their non-infected counterparts. This is consistent with institutional and multicenter reports of cardiac arrest among COVID-19 patients, which have reported survival rates ranging from 0% to 21% [22,25,26]. In a single center study of 136 patients in Wuhan, China, only 1 of 136 COVID-19 patients who experienced in-hospital cardiac arrest had a favorable neurologic outcome following cardiac arrest [27]. Interestingly, in our analysis, cardiac arrest patients with COVID-19 had greater odds of ECMO utilization relative to other cardiac arrest patients. This finding may, in part, be related to the time frame selected for study, as limited data was available in 2020 regarding short- and mid-term survival of patients with severe COVID-19 infection. Based on these series, the Extracorporeal Life Support Organization cautions against extracorporeal CPR in COVID-19 patients [28], which is similarly supported by the results of our study.

### Limitations

The present study has several limitations related to its retrospective design and the nature of the National Inpatient Sample. Cardiac arrest was identified using ICD-10 codes and limited data is available regarding the duration of CPR, medications administered during cardiac arrest, and specific location of arrest, which may influence outcomes. As such, our study captures those who require venoarterial extracorporeal support and should not be extrapolated to venovenous support for respiratory failure. Furthermore, the NIS only captures patients who survive to hospital admission, and, thus, our study likely overestimates the survival rate of all patients experiencing out of hospital cardiac arrest, as those who died prior to arrival at a facility were not captured. In addition, the severity of COVID-19 infection could not be ascertained as laboratory values and imaging findings are not available for study in the NIS. We limited our analysis to data through 2020, as we wished to evaluate outcomes immediately during the COVID-19 pandemic period. Further temporal study of cardiac arrest outcomes following the COVID-19 pandemic may yield insights regarding national healthcare recovery from the COVID-19 pandemic. Nonetheless, our analysis utilized robust statistical methodology and the largest all-payer database in the United States to derive nationally representative observations.

### Conclusion

In conclusion, cardiac arrest incidence and mortality significantly increased during the COVID-19 Pandemic, contrary to improvements in cardiac arrest survival over the last decade. ECMO utilization for cardiac arrest initially declined, though mortality among ECMO patients remained elevated. These findings emphasize the need for efforts to evaluate and improve care of cardiac arrest and ECMO patients nationally. COVID-19 patients who experienced cardiac arrest had dismal outcomes relative to non-COVID-19 patients, suggesting no role for ECMO in this patient population.

## Supporting information

S1 Table International classification of diseases, tenth revision (ICD-10) codes for identification of procedures and diagnoses. (DOCX)

S2 Table Adjusted multivariable model for mortality among non-COVID-19 cardiac arrest patients treated from 2016–2020. PEA, pulseless electrical activity; VF, ventricular fibrillation; VT, ventricular tachycardia.
(DOCX)

S3 Table Adjusted multivariable model for use of extracorporeal membrane oxygenation among non-COVID-19 cardiac arrest patients treated from 2016–2020. PEA, pulseless electrical activity; VF, ventricular fibrillation; VT, ventricular tachycardia.
(DOCX)
