# Supplementary material for: Trends and outcomes of cardiac arrest and extracorporeal membrane oxygenation during the COVID-19 pandemic in the United States
Source: PLoS One. 2025 Oct 16;20(10):e0334896. doi: 10.1371/journal.pone.0334896 (PMC12530557; doi:10.1371/journal.pone.0334896)
Supplement: S1 Table — (DOCX) [file pone.0334896.s001.docx]

**S1 Table. International Classification of Diseases, Tenth Revision (ICD-10) codes for identification of procedures and diagnoses.**

| **Diagnosis/Procedure** | **ICD-10 Codes** |
| --- | --- |
| Cardiac Arrest Setting |  |
| Out-of-Hospital | I46.2, I46.8, I46.9 |
| In-Hospital | 5A12012, 5A1221Z |
| ECMO | 5A1522F, 5A1522G, 5A1522H |
| COVID-19 | J12.82, U00, U07.1, U09.9, U49, U50, U85 |
