# Supplementary material for: Trends and outcomes of cardiac arrest and extracorporeal membrane oxygenation during the COVID-19 pandemic in the United States
Source: PLoS One. 2025 Oct 16;20(10):e0334896. doi: 10.1371/journal.pone.0334896 (PMC12530557; doi:10.1371/journal.pone.0334896)
Supplement: S2 Table — PEA, pulseless electrical activity; VF, ventricular fibrillation; VT, ventricular tachycardia. (DOCX) [file pone.0334896.s002.docx]

**S2 Table. Adjusted multivariable model for mortality among non-COVID-19 cardiac arrest patients treated from 2016-2020.** PEA, pulseless electrical activity; VF, ventricular fibrillation; VT, ventricular tachycardia.

|  | Adjusted odds ratio | 95% confidence interval | P-value |
| --- | --- | --- | --- |
| Age (1-year increment) | 1.02 | 1.02 - 1.03 | <0.001 |
| Female (ref: Male) | 1.03 | 1.01 – 1.05 | 0.001 |
| Income Quartile |  |  | <0.001 |
| 0-25^th^ Percentile | Reference |  |  |
| 26-50^th^ Percentile | 0.94 | 0.92 – 0.97 |  |
| 51-75^th^ Percentile | 0.88 | 0.86 – 0.90 |  |
| 76-100^th^ Percentile | 0.85 | 0.83 – 0.88 |  |
| Race |  |  | <0.001 |
| White | Reference |  |  |
| Black | 1.09 | 1.06 – 1.12 |  |
| Hispanic | 1.12 | 1.08 – 1.16 |  |
| Asian and Pacific Islander | 1.21 | 1.14 – 1.27 |  |
| Other race | 1.16 | 1.10 – 1.21 |  |
| Insurance Type |  |  | <0.001 |
| Private | Reference |  |  |
| Medicare | 1.14 | 1.14 – 1.17 |  |
| Medicaid | 1.22 | 1.18 – 1.26 |  |
| Other payer | 1.19 | 1.12 – 1.27 |  |
| Uninsured | 1.85 | 1.76 – 1.94 |  |
| Comorbidities |  |  |  |
| Cerebrovascular disease | 0.97 | 0.94 – 0.99 | 0.02 |
| Chronic pulmonary disease | 1.05 | 1.03 – 1.07 | <0.001 |
| Cirrhosis | 2.01 | 1.91 – 2.11 | <0.001 |
| Congestive heart failure | 0.68 | 0.67 – 0.70 | <0.001 |
| End-stage renal disease | 1.03 | 1.00 – 1.05 | 0.04 |
| Malignancy | 1.77 | 1.71 – 1.83 | <0.001 |
| Myocardial infarction | 0.85 | 0.83 – 0.86 | <0.001 |
| Pulmonary circulation disorders | 0.95 | 0.92 – 0.98 | 0.004 |
| Pulmonary embolism | 1.16 | 1.10 – 1.22 | <0.001 |
| Elixhauser Comorbidity Index  (1-point increment) | 1.01 | 0.99 – 1.01 | 0.05 |
| Arrest Location |  |  |  |
| In-hospital cardiac arrest | 1.72 | 1.69 – 1.76 | <0.001 |
| Out-of-hospital cardiac arrest | Reference |  |  |
| Rhythm |  |  |  |
| VT or VF | 0.55 | 0.54 – 0.56 | <0.001 |
| Asystole or PEA | Reference |  |  |
| Hospital Characteristics (%) |  |  |  |
| Bed size |  |  |  |
| Small | Reference |  |  |
| Medium | 1.02 | 0.99 – 1.05 | 0.22 |
| Large | 1.03 | 1.00 – 1.06 | 0.08 |
| Region |  |  |  |
| Northeast | Reference |  |  |
| Midwest | 0.85 | 0.83 – 0.89 | <0.001 |
| South | 0.91 | 0.88 – 0.94 | <0.001 |
| West | 0.99 | 0.96 – 1.03 | 0.70 |
| Pandemic | 1.16 | 1.12 – 1.19 | <0.001 |
