# Supplementary material for: Trends and outcomes of cardiac arrest and extracorporeal membrane oxygenation during the COVID-19 pandemic in the United States
Source: PLoS One. 2025 Oct 16;20(10):e0334896. doi: 10.1371/journal.pone.0334896 (PMC12530557; doi:10.1371/journal.pone.0334896)
Supplement: S3 Table — PEA, pulseless electrical activity; VF, ventricular fibrillation; VT, ventricular tachycardia. (DOCX) [file pone.0334896.s003.docx]

**S3 Table. Adjusted multivariable model for use of extracorporeal membrane oxygenation among non-COVID-19 cardiac arrest patients treated from 2016-2020.** PEA, pulseless electrical activity; VF, ventricular fibrillation; VT, ventricular tachycardia.

|  | Adjusted odds ratio | 95% confidence interval | P-value |
| --- | --- | --- | --- |
| Age (1-year increment) | 0.96 | 0.95 – 0.96 | <0.001 |
| Female (ref: Male) | 0.89 | 0.81 – 0.97 | 0.007 |
| Income Quartile |  |  | <0.001 |
| 0-25^th^ Percentile | Reference |  |  |
| 26-50^th^ Percentile | 1.36 | 1.20 – 1.55 |  |
| 51-75^th^ Percentile | 1.46 | 1.28 – 1.67 |  |
| 76-100^th^ Percentile | 1.95 | 1.68 – 2.26 |  |
| Race |  |  | <0.001 |
| White | Reference |  |  |
| Black | 0.89 | 0.78 – 1.01 |  |
| Hispanic | 1.17 | 0.99 – 1.37 |  |
| Asian and Pacific Islander | 1.11 | 0.87 – 1.43 |  |
| Other race | 1.27 | 1.01 – 1.59 |  |
| Insurance Type |  |  |  |
| Private | Reference |  |  |
| Medicare | 0.49 | 0.43 – 0.56 | <0.001 |
| Medicaid | 0.56 | 0.49 – 0.63 | <0.001 |
| Other payer | 0.72 | 0.57 – 0.91 | 0.006 |
| Uninsured | 0.51 | 0.41 – 0.63 | <0.001 |
| Comorbidities |  |  |  |
| Cerebrovascular disease | 0.75 | 0.63 – 0.91 | 0.003 |
| Chronic pulmonary disease | 0.49 | 0.44 – 0.56 | <0.001 |
| Cirrhosis | 0.34 | 0.24 – 0.49 | <0.001 |
| Congestive heart failure | 1.87 | 1.66 – 2.10 | <0.001 |
| End-stage renal disease | 0.40 | 0.33 – 0.48 | 0.04 |
| Malignancy | 0.35 | 0.28 – 0.44 | <0.001 |
| Myocardial infarction | 1.36 | 1.22 – 1.51 | <0.001 |
| Pulmonary circulation disorders | 1.45 | 1.25 – 1.69 | <0.001 |
| Pulmonary embolism | 1.94 | 1.61 – 2.33 | <0.001 |
| Elixhauser Comorbidity Index  (1-point increment) | 1.19 | 1.16 – 1.22 | <0.001 |
| Arrest Location |  |  |  |
| In-hospital cardiac arrest | 1.67 | 1.51 – 1.85 | <0.001 |
| Out-of-hospital cardiac arrest | Reference |  |  |
| Rhythm |  |  |  |
| VT or VF | 1.56 | 1.41 – 1.72 | <0.001 |
| Asystole or PEA | Reference |  |  |
| Hospital Characteristics |  |  |  |
| Bed size |  |  |  |
| Small | Reference |  |  |
| Medium | 1.09 | 0.85 – 1.39 | 0.52 |
| Large | 3.64 | 2.93 – 4.53 | <0.001 |
| Region |  |  |  |
| Northeast | Reference |  |  |
| Midwest | 0.76 | 0.63 – 0.92 | 0.005 |
| South | 0.66 | 0.55 – 0.79 | <0.001 |
| West | 0.44 | 0.36 – 0.55 | <0.001 |
| Pandemic | 0.99 | 0.85 – 1.16 | 0.90 |
